# Could Alcohol-Related Cognitive Decline Be the Result of Iron-Induced Neuroinflammation?

**DOI:** 10.3390/brainsci14060520

**Published:** 2024-05-21

**Authors:** Thomas D. W. Wilcockson, Sankanika Roy

**Affiliations:** 1 School of Sport, Exercise and Health Sciences, Loughborough University, Loughborough LE11 3TU, UK; 2 Department of Neurology, Leicester Royal Infirmary, Leicester LE1 5WW, UK; sr582@leicester.ac.uk

**Keywords:** alcohol, iron accumulation, neuroinflammation, cognitive decline, neurodegeneration, oxidative stress, APOE4, Alzheimer’s disease

## Abstract

Excessive and prolonged alcohol use can have long-term severe neurological consequences. The mechanisms involved may be complicated; however, new evidence seems to indicate the involvement of iron accumulation and neuroinflammation. Prolonged alcohol consumption has been linked to the accumulation of iron in specific regions of the brain. Evidence suggests that excess iron in the brain can trigger microglia activation in response. This activation leads to the release of pro-inflammatory cytokines and reactive oxygen species, which can cause damage to neurons and surrounding brain tissue. Additionally, iron-induced oxidative stress and inflammation can disrupt the blood–brain barrier, allowing immune cells from the periphery to infiltrate the brain. This infiltration can lead to further neuroinflammatory responses. Inflammation in the brain subsequently disrupts neuronal networks, impairs synaptic plasticity, and accelerates neuronal cell death. Consequently, cognitive functions such as memory, attention, and decision-making are compromised. Additionally, chronic neuroinflammation can hasten the development and progression of neurodegenerative diseases, further exacerbating cognitive impairment. Therefore, alcohol could act as a trigger for iron-induced neuroinflammation and cognitive decline. Overall, the mechanisms at play here seem to strongly link alcohol with cognitive decline, with neuroinflammation resulting from alcohol-induced iron accumulation playing a pivotal role.

## 1. Introduction

Excessive and prolonged alcohol use can have long-term severe neurological consequences ranging from short-term memory impairment [1] to debilitating cognitive decline [2]. In fact, even moderate drinking has well-documented detrimental effects on the central nervous system and may be associated with cognitive decline [3]. Therefore, alcohol is considered a modifiable risk factor which, if controlled, could reduce the likelihood of cognitive decline [4]. There is some debate about just how damaging alcohol can be, with some authors suggesting alcohol can have protective qualities [5]. However, the overwhelming majority of evidence suggests alcohol has a detrimental effect [6]. Indeed, a large-scale longitudinal analysis of 31 million hospital admissions revealed a significant association between alcohol use disorders (addiction or harmful use as defined in ICD) and increased dementia risk [7]. Notably, the connection between alcohol use disorders and dementia was particularly strong in younger-onset cases (below 65 years old), where over half of those diagnosed had documented alcohol abuse. One comprehensive analysis found a clear association between the amount of alcohol consumed and the likelihood of developing dementia [8]. Their findings revealed a significant increase in dementia risk with heavy drinking (≥23 drinks/week or ≥38 g/day). Additionally, a U.K. biobank study of 13,342 middle-aged volunteers (40–73 years old) reported a decline in reaction time for those consuming more than 12 units per week, compared with lighter drinkers. At follow-up, drinking more than 21 units per week was associated with a 17% (95% CI 4–32 and 13–23, respectively) increase in dementia compared with drinking less than 14 units. Heavy drinking was also associated with shrinkage in the right hippocampus, a brain region crucial for memory, as observed by MRI scans [3,9]. It is worth noting that both Xu et al. [8] and Piumatti et al. [9] found evidence that light drinking may in fact have protective qualities over complete abstinence. However, these findings may be biased by the unclear nature of alcohol abstainers. For example, a U.K. Whitehall study with over 9000 participants tracked for 23 years identified both heavy drinking (over 21 units per week) and long-term abstinence as potential risk factors for dementia, compared with consuming less than 14 units [10]. However, although these results may demonstrate a detrimental effect of long-term abstinence, it is unclear whether those reporting abstinence are abstinent because they suspect cognitive impairment already, and so reduce their alcohol intake, or if they are long-term abstinent because of previously having engaged too heavily in alcohol use. In essence, there are myriad reasons why someone may choose to abstain from alcohol, and some of those reasons may themselves have an effect on cognitive decline. However, the results may indicate that some people are more at risk of cognitive decline as a result of alcoholism than others. The aim of this literature review is to explore the relationship between alcohol-induced iron accumulation, neuroinflammation, and the effects of this on cognitive decline.

## 2. Alcohol-Induced Iron Accumulation in the Brain

Previous findings potentially highlight a complex and multifaceted relationship between the quantity of alcohol consumed and dementia risk. Heavy drinking undoubtedly poses a significant threat and understanding the mechanisms underlying the neurotoxic effects of alcohol is nevertheless important. The neurobiological mechanisms underlying alcohol-induced neurological impairments are likely multifaceted, involving disruption of neurotransmitter systems, neuroinflammation, oxidative stress, and alterations in neuroplasticity. Alcohol disrupts synaptic transmission by inhibiting the release of the neurotransmitter acetylcholine in the cerebral cortex [11] via inhibition of voltage-gated calcium channels [12]. Alcohol also inhibits the N-methyl-d-aspartic acid subtype of glutamate receptors, thus disrupting the flow of several ions across the cell membranes in the hippocampal neurons [13]. Disrupted neurotransmission further plays a major role in cognitive decline, as this neuroinflammation induced by alcohol intake may also play a major role in cognitive dysfunction [14]. Several studies have been performed to explain the underlying pathophysiology. Alcohol-induced microglial activation plays a major role in neuronal inflammatory responses [15,16]; the neuroinflammatory processes include activation of the neuroinflammatory mediators like Toll-like receptor 4 (TLR4) signalling and Interleukin-1beta (IL-1β) and Interleukin-18 (IL-18) cytokine production [16]. In addition to these, a recent study has shown that chronic alcohol intake leads to the accumulation of iron in the brain [17], playing a major role in cognitive decline. This finding is significant, as previous research has indicated that intra-cellular iron buildup in the brain may contribute to neuroinflammation [18]. As neuroinflammation is associated with cognitive decline, these findings present an intriguing hypothesis regarding a potential mechanism for how alcohol can contribute toward cognitive decline.

Iron is an important micronutrient and plays a critical role in metabolic processes, including oxygen transport through haemoglobin, oxidative phosphorylation in the cytochromes via the electron transport chain [19], and the immune response. The normal iron content of the male adult body is 35 to 45 mg/kg body weight [20]. The liver parenchymal and reticulo-endothelial cells are the storehouses of iron. However, excessive iron in the body is potentially toxic to cells. Iron takes part in the Fenton reaction [21], resulting in the generation of hydroxyl radicals as well as higher oxidation states of iron, causing severe biological damage. Iron overload amplifies the damaging effects of superoxides in a wide array of inflammatory reactions as well [22]. Prolonged and even moderate alcohol consumption has been implicated in excess iron accumulation in the body, leading to iron buildup in specific brain regions like the basal ganglia and hippocampus [17]. Topiwala et al. [17] found that the accumulation of iron in the brain observed in moderate drinkers provides evidence of higher putamen and caudate iron levels, which were previously described by Juhás, et al. (2017) [23]. However, whereas Juhas’ samples drank excessive amount (>37 units daily), Topiwala observed such associations in those who drank a moderate amount of just >7 units per week. These data therefore demonstrate that even moderate amounts of prolonged alcohol usage can lead to iron accumulation in the brain. Additionally, Wang et al. [24] also observed that iron accumulates in basal ganglia regions with higher frequency binge drinking. The link between alcohol and iron accumulation in the brain is likely intricate, involving a multitude of direct and indirect effects on iron metabolism. Alcohol can directly impact iron absorption in the gut by increasing intestinal permeability, leading to increased iron levels in the blood [25]. Additionally, animal studies have shown that any inflammatory stimuli can increase the expression of iron transporters DMT1 and ferroprotein in neurons, astrocytes, and microglia, and it also increases the expression of the iron regulator in hepcidin in microglia and astrocytes [26]. Chronic alcohol consumption disrupts the integrity of the blood–brain barrier, a protective layer that separates the brain from circulating blood [27]. A compromised blood–brain barrier allows greater iron flow into the brain, setting the stage for localised accumulation [28]. Within the brain, alcohol exacerbates oxidative stress, a state of imbalance between harmful free radicals and the body’s antioxidant defences [29]. This increase in oxidative stress promotes the release of iron from its stores, leading to its buildup in brain tissue [30]. Furthermore, alcohol-induced inflammation within the brain can further drive iron dysregulation, ultimately contributing to neuronal damage and the potential for cognitive impairments [16]. Therefore, even moderate alcohol intake disrupts iron homeostasis through multiple pathways, leading to potentially neurotoxic iron buildup in the brain.

## 3. Iron Accumulation in the Brain and Neuroinflammation

Emerging evidence suggests that elevated iron levels in the brain trigger the activation of microglia, the brain’s resident immune cells [18]. When encountering excess iron, microglia transform into a pro-inflammatory state, releasing harmful molecules like pro-inflammatory cytokines and reactive oxygen species [18]. These molecules can damage surrounding neurons and brain tissue. However, this response, while intended to be protective, can have unintended collateral damage. Certain pro-inflammatory cytokines, like interleukin-1β (IL-1β), tumour necrosis factor-alpha (TNF-α), and interleukin-6 (IL-6), possess potent neurotoxic properties [31]. They instigate a state of neuroinflammation, characterised by chronic low-grade inflammation within the brain. This inflammation, similar to the body’s response to an infection, involves the recruitment of additional immune cells and the production of various harmful molecules, including reactive oxygen species [32]. Alcohol increases the activity of the liver enzyme cytochrome P450, which metabolises alcohol to generate reactive oxygen species, and alcohol’s ability to increase iron accumulation further promotes reactive oxygen species generation [33]. The most reactive oxygen species is the hydroxyl radical, which is generated by the reaction between superoxide radicals and hydrogen peroxide. Under normal physiological conditions, this interaction is not possible, but in the presence of free iron, this reaction is feasible, resulting in the production of hydroxyl radicals [33]. Reactive oxygen species are highly reactive molecules containing oxygen that can inflict widespread damage on surrounding cells as they are capable of binding to most macromolecule proteins, lipids, and even the DNA. They can directly attack the delicate membranes of neurons, leading to their dysfunction and, ultimately, death. This ongoing assault on neurons by both pro-inflammatory cytokines and reactive oxygen species contributes significantly to the neurodegeneration observed in various neurological conditions, including those associated with chronic alcohol consumption and iron overload [26]. Furthermore, sustained neuroinflammation disrupts the delicate balance of communication among neurons, hindering their ability to transmit signals effectively [34]. This, in turn, can manifest as cognitive decline, memory impairment, and other neurological symptoms often associated with alcohol-related brain damage. Additionally, iron-induced oxidative stress and inflammation can disrupt the blood–brain barrier [35], allowing immune cells from the periphery to infiltrate the brain. This infiltration can lead to further neuroinflammatory responses.

The blood–brain barrier is an intricate network of specialised endothelial cells that lines the brain’s blood vessels and forms a barrier. This structure protects and regulates the passage of molecules and cells between the bloodstream and the brain’s delicate environment. Alcohol-induced thiamine deficiency has been proposed to disrupt the blood–brain barrier [35]. This weakening of the blood–brain barrier allows for the infiltration of immune cells from the periphery of the body into the previously protected brain tissue. These immune cells, primarily monocytes and lymphocytes, are normally recruited to fight off infections and clear debris outside the brain. However, their presence within the brain parenchyma, because of a compromised blood–brain barrier, can exacerbate the ongoing neuroinflammatory response [36]. Once inside the brain, these infiltrated immune cells interact with activated microglia, further amplifying the inflammatory cascade. They release additional pro-inflammatory cytokines and reactive oxygen species, contributing to the ongoing neuronal damage. Additionally, these cells can directly attack healthy neurons, mistaking them for foreign invaders, leading to further tissue destruction and functional decline. Moreover, the presence of these peripheral immune cells within the brain can trigger the activation of astrocytes, another type of glial cell. Activated astrocytes, in turn, can contribute to the inflammatory response by producing additional inflammatory mediators and forming scar tissue, further hindering communication among neurons and impeding repair processes. This influx of immune cells, facilitated by a compromised blood–brain barrier, creates a vicious cycle of inflammation within the brain.

## 4. Neuroinflammation and Cognitive Decline

Iron-induced inflammation in the brain causes a cascade of events that disrupt brain function by affecting synapses. Pro-inflammatory cytokines and reactive oxygen species disrupt the balance of chemical messengers at the synapse, hindering the ability of neurons to transmit signals effectively [37]. This impaired synaptic plasticity, the brain’s ability to adapt and form new connections, translates into a gradual decline in cognitive function. Furthermore, the chronic inflammatory environment created by iron overload and immune cell infiltration directly impacts the vitality and viability of neurons. Pro-inflammatory molecules and reactive oxygen species can therefore trigger the activation of apoptosis [38]. The loss of neurons, coupled with the disruption of synaptic connections, has a profound impact on brain function. This translates into a decline in cognitive abilities, including memory, learning, attention, and executive function [39]. Indeed, this cascade disrupts neuronal networks, impairs synaptic plasticity, and accelerates neuronal death, ultimately leading to a progressive decline in cognitive function.

The initial impact of this neurodegenerative process often manifests as subtle changes in cognitive abilities. Individuals may experience difficulties with memory, particularly short-term memory, making it challenging to remember recent events or conversations [40]. Executive function also becomes compromised. Individuals might struggle with prospective memory, prioritising tasks, or making decisions [41]. As neuroinflammation progresses, attention and focus become increasingly challenging [42]. The ability to filter out distractions and maintain concentration on a single task deteriorates, leading to reduced productivity and difficulty completing everyday tasks. Additionally, language skills may be affected, manifesting as difficulties with word retrieval, fluency, or comprehension [43]. In severe cases, the cumulative effects of neuronal loss and disrupted communication networks can lead to more significant cognitive decline [44]. The specific cognitive functions affected and the severity of decline can vary depending on several factors, including the individual’s age, sex, genetic predispositions, and the duration and severity of alcohol abuse [45]. However, the underlying mechanism—the neuroinflammatory cascade triggered by iron accumulation—may be a crucial factor in understanding the devastating effects of chronic alcohol consumption on brain health and cognitive function. Overall, evidence suggests the importance of iron homeostasis for brain function, and disruption of this balance can lead to neurodegenerative diseases [24]. This has led to the “ferroptosis hypothesis” of Alzheimer’s disease. Zhang et al. suggest that when iron levels are imbalanced, the antioxidant system in the brain is disrupted, which leads to oxidative stress and cell death [46]. It is therefore proposed that ferroptosis, a specific type of cell death caused by iron overload, may be a major factor in Alzheimer’s disease. These findings suggest a potential therapeutic approach, whereby targeting ferroptosis may prevent Alzheimer’s disease. Therefore, progressive neuroinflammation, triggered by iron accumulation, appears to be a central mechanism underlying the effects of chronic alcohol abuse, which may ultimately lead to cognitive decline.

With the decline in cognitive function, continued chronic neuroinflammation can hasten the development and progression of neurodegenerative diseases, further exacerbating cognitive impairment. Neurodegenerative diseases like Alzheimer’s disease, Parkinson’s disease, and Amyotrophic Lateral Sclerosis are characterised by the progressive loss of neurons and the accumulation of abnormal protein aggregates within the brain [37]. These protein aggregates, such as amyloid plaques and tau tangles in Alzheimer’s disease, are believed to play a crucial role in the disease process [47]. Some evidence suggests that chronic neuroinflammation may create an environment that enhances the development and progression of these protein aggregates as the pro-inflammatory cytokines and reactive oxygen species directly damage neurons, making them more susceptible to the toxic effects of these protein aggregates [48]. Additionally, the inflammatory environment disrupts the brain’s natural waste clearance mechanisms, allowing these harmful protein aggregates to accumulate further. Furthermore, chronic neuroinflammation may disrupt signalling pathways within the brain. These pathways are crucial for maintaining neuronal health and promoting the survival and function of neurons [49]. When disrupted by inflammation, these pathways can contribute to increased neuronal vulnerability and accelerate the neurodegenerative process. The combined effects of direct neuronal damage, enhanced protein aggregation, and disrupted signalling pathways by chronic neuroinflammation ultimately accelerate the development and progression of neurodegenerative diseases. This further exacerbates cognitive impairment, leading to a more rapid decline in memory, learning, attention, and executive function.

The detrimental effects of chronic alcohol consumption on brain health, mediated by iron-induced neuroinflammation, may be amplified in individuals carrying the Apolipoprotein E ε4 allele (APOE4). This specific genetic variant has been linked to increased susceptibility to neuroinflammation in Alzheimer’s disease [50]. Studies have shown that individuals with APOE4 exhibit higher levels of iron accumulation within brain regions critical for memory and cognition [51]. APOE4 plays a crucial role in cholesterol transport and metabolism within the brain. Unfortunately, the APOE4 variant has been shown to be less efficient in clearing harmful cholesterol deposits, leading to a condition known as cerebral amyloid angiopathy. Cerebral amyloid angiopathy is characterised by the build-up of abnormal amyloid protein deposits within the walls of the blood vessels of the brain. This compromised clearance of cholesterol and the presence of cerebral amyloid angiopathy in individuals with APOE4 are believed to contribute to a pro-inflammatory environment within the brain, making them more susceptible to the detrimental effects of iron overload. Additionally, abnormal amyloid deposits in vessel walls make them very fragile and manifest in lobar intracerebral haemorrhage, resulting in acute haemorrhagic strokes [52]. Chronic alcohol consumption, by further disrupting iron metabolism and triggering neuroinflammation, can exacerbate this vulnerability in APOE4 carriers. The combined effects of APOE4, iron overload, and chronic alcohol exposure may create a synergistic detrimental impact on brain health. This can lead to a faster progression of neurodegenerative diseases like Alzheimer’s disease, particularly for individuals carrying the APOE4 allele.

## 5. Discussion

**Figure 1:**
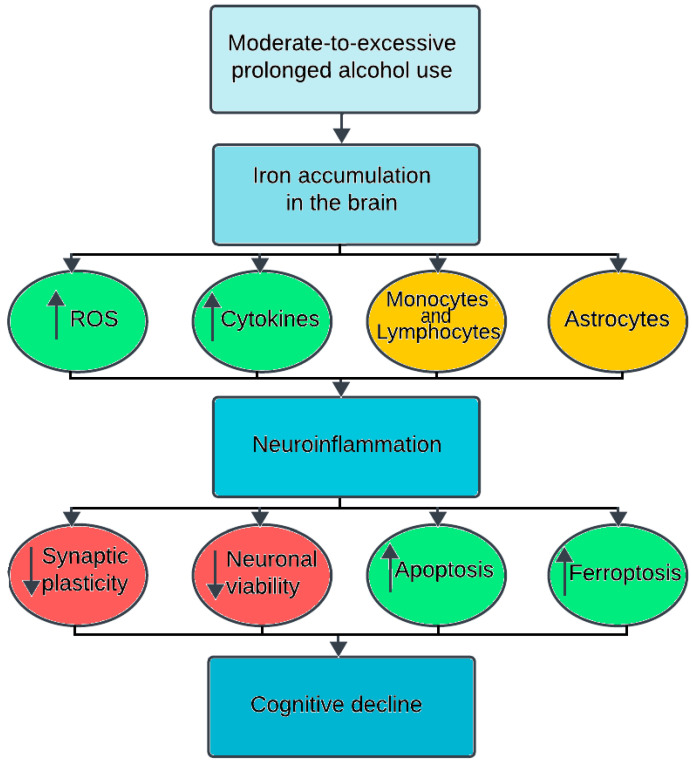
Schematic representation of alcohol-induced iron accumulation in the brain leading to neuroinflammation followed by cognitive decline. Alcohol intake increases the iron load in the brain, resulting in the activation of reactive oxygen species (ROS), cytokines, monocytes and lymphocytes influx, and the activation of astrocytes, leading to neuroinflammation that causes a reduction in neuronal plasticity and synaptic transmission and increased neuronal death, altogether resulting in cognitive decline. Note, arrows pointing up denotes an increase, while an arrow pointing down denotes a decrease.

**Figure 2:**
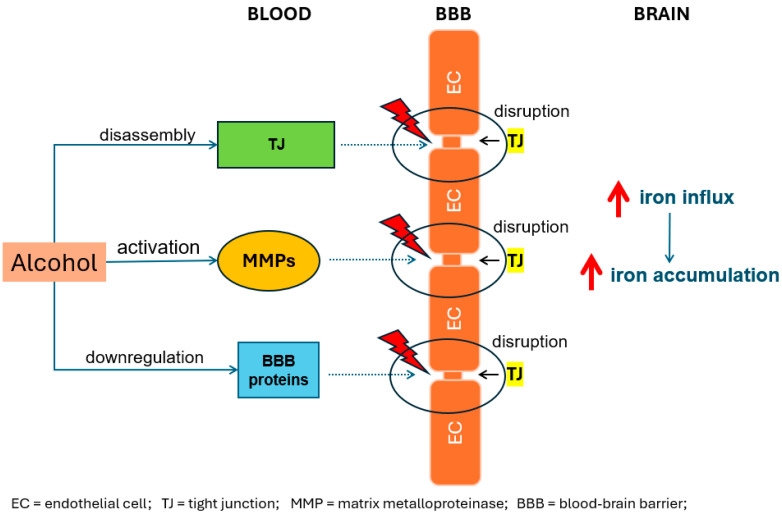
Alcohol disrupts the blood–brain barrier (BBB) by means of disrupting tight junctions (TJs) either via downregulation of TJ proteins or activation of matrix metalloproteinase (MMP), which can directly cause TJ breakdown. Increased iron influx leads to increased iron accumulation.

Overall, the mechanisms at play here seem to strongly link alcohol with cognitive decline, with neuroinflammation resulting from alcohol-induced iron accumulation playing a pivotal role. While the definitive cause behind alcohol-induced cognitive decline remains under investigation, a compelling narrative emerges, implicating iron-induced neuroinflammation as a potential mediator in this detrimental process. This intricate cascade unfolds in several key steps as follows: chronic alcohol consumption disrupts iron metabolism, leading to its overload in specific brain regions (see Figure 1). This excess iron acts as a source of oxidative stress and neurotoxicity. Furthermore, chronic alcohol exposure weakens the blood–brain barrier [53], allowing harmful substances, including excess iron, to infiltrate the brain and exacerbate the detrimental effects (see Figure 2). Carreno et al. showed that alcohol caused endothelial cell tight-junction disassembly, leading to gaps in the blood–brain barrier in rat brain models [53]; another study showed alcohol-induced down-regulation of blood–brain barrier proteins in the frontal cortex of animal models [54]. In addition to this, several post-mortem studies have shown a disrupted blood–brain barrier in individuals with alcohol use disorder [55], whilst other in vitro model studies have shown that alcohol can damage the blood–brain barrier by activating matrix metalloproteinases and disrupting the basement membrane [56]. In response to the iron overload, microglia, the brain’s resident immune cells, become activated in an attempt to clear the excess iron. However, this activation leads microglia to release pro-inflammatory cytokines and reactive oxygen species. While intended as a defence mechanism, these inflammatory mediators inflict collateral damage on surrounding neurons, disrupting their function and ultimately leading to impaired cognitive abilities like memory, attention, and executive function. This complex interplay among alcohol, iron, and neuroinflammation provides a crucial piece of the puzzle in understanding the potential mechanisms behind alcohol-related cognitive decline.

There are many possible directions for future research related to this. While evidence suggests a possible connection, more research is needed to establish a definitive causal link between iron-induced neuroinflammation and alcohol-related cognitive decline. Iron buildup in the brain could potentially impair executive function and fluid intelligence. Additionally, chronic heavy alcohol consumption is known to cause frontal lobe dysfunction [57]. Studies have also shown a possible link between high dietary iron intake and decreased cognitive function [58]. Furthermore, iron has been observed to accumulate alongside tau and beta-amyloid proteins in the brain, both of which are associated with Alzheimer’s disease [59]. Moreover, iron can trigger cell death through apoptosis and ferroptosis [60], potentially contributing to neurodegeneration. Ferroptosis is a type of programmed cell death dependent on iron, and excess iron leads to lipid peroxidation, glutathione peroxidase 4 enzyme activity, and the metabolism of iron, all leading to neuronal cell death and degeneration [61]. Overall, evidence suggests that iron accumulation in the brain is associated with poorer cognitive outcomes. Whether this is directly related to neuroinflammation is still unknown. Future studies employing longitudinal designs and experimental models could provide stronger evidence for causality. Further, a deeper understanding of the specific mechanisms by which iron overload triggers neuroinflammation and disrupts neuronal function is needed. This could involve investigating the role of microglial activation, pro-inflammatory cytokines, and other signalling pathways involved in the neurodegenerative process. The potential synergistic effects of genetic predispositions, like the APOE4 allele, on iron-induced neuroinflammation and cognitive decline warrant further investigation. Studies exploring the interplay among genetic factors, iron metabolism, and alcohol consumption could provide valuable insights into individual risk profiles. Other factors such as age, sex, dietary habits, and pre-existing health conditions may also influence individual susceptibility to the detrimental effects of alcohol and iron overload on brain health. Investigating these factors is crucial for developing personalised prevention and intervention strategies. Finally, translating the growing understanding of iron-induced neuroinflammation into effective therapeutic and preventative strategies could be fruitful. This could involve exploring chelation therapy to remove excess iron from the brain [62], developing anti-inflammatory drugs targeted specifically for the brain, and investigating dietary and lifestyle modifications that can mitigate iron accumulation and neuroinflammation. While further research is clearly needed, the current evidence suggests a strong plausible connection among alcohol-related iron overload, neuroinflammation, and cognitive decline. Further research focused on establishing causality, understanding individual differences, and translating findings into practical interventions has the potential to significantly improve brain health outcomes, particularly for individuals struggling with chronic alcohol consumption or at higher risk because of genetic predispositions. By unravelling the intricate relationship among these factors, we can pave the way for a future where brain health is protected and cognitive decline associated with alcohol consumption is effectively mitigated.

Alcohol-induced iron overload in the brain currently lacks pharmacological management in humans. There have been studies in animal models that have suggested successful pharmacological intervention by reducing iron overload or iron-induced pathology in mice models. One study showed that treatment with deferoxamine could reduce tau-phosphorylation in transgenic mice models [63]. A recent review involving two iron chelating agents, M30 and HLA20, suggested the neuroprotective role of iron-chelating agents in animal models by reducing neurodegenerative pathology, upregulating signalling pathways that provide neuronal protection, and providing positive behavioural modification [37]. Several studies have recently proposed the utilisation of iron-chelating agents for the treatment of Alzheimer’s disease [62,64]. Another study used alpha-lipoic acid in transgenic mice models, which prevented tau-hyperphosphorylation and reduced oxidative stress, inflammation, and ferroptosis in neuronal cells [65]. Thiamine has been proposed to prevent brain iron overload [35]. Alcohol-induced thiamine deficiency has been suggested to play a major role in increasing blood–brain barrier permeability, leading to the increased influx of iron into the brain. Thiamine replacement plays a major role in alcohol use disorder management, and it can also be utilised for the prevention of iron overload in the brain. However, more research and clinical trials are required to explore the avenues for pharmaceutical management of brain iron accumulation and iron-induced cognitive decline.

## 6. Conclusions

In conclusion, excessive prolonged alcohol consumption could act as a trigger for iron-induced neuroinflammation and subsequent cognitive decline. Although it is difficult to quantify “excess”, the most recent data suggest that alcohol consumption above seven units per week leads to high iron accumulation in the brain [17]. Excess iron in the brain becomes harmful, damaging brain cells and compromising their function. Additionally, alcohol weakens the blood–brain barrier, allowing further infiltration of harmful substances, including the additional excess iron. Microglia attempting to clear the iron may inadvertently cause further damage through the release of inflammatory molecules. These molecules directly attack neurons, disrupt learning and adaptation abilities, and contribute to the accumulation of harmful protein aggregates linked to neurodegenerative diseases. People with the APOE4 allele may be especially at risk. By understanding this complex interplay among alcohol, iron, and neuroinflammation, researchers can develop strategies to mitigate these detrimental effects and potentially protect brain health in the face of alcohol misuse.
